# Psycho-Physiological Associates of Dyspnea in Hospitalized Patients with Interstitial Lung Diseases: A Cross-Sectional Study

**DOI:** 10.3390/ijerph14101277

**Published:** 2017-10-24

**Authors:** Yan Hua Zhou, Yim Wah Mak

**Affiliations:** 1 School of Nursing, Faculty of Health and Social Sciences, The Hong Kong Polytechnic University, Hung Hom, Kowloon, Hong Kong 999077, China; 15098644g@connect.polyu.hk; 2 School of Nursing, Zhejiang Chinese Medical University, Hangzhou 310053, China

**Keywords:** dyspnea, interstitial lung diseases, psycho-physiological factors, cough, six-minute walk test, anxiety, depression, lung function

## Abstract

Dyspnea has been found to be an independent predictor of mortality among patients with respiratory diseases and is often regarded as a difficult symptom to control in patients with interstitial lung diseases (ILDs). Previous studies have found an association of psychological and physiological factors with dyspnea among patients with chronic obstructive airway diseases. However, symptom management of hospitalized patients with ILDs has been hampered by difficulty in priority, since they are often admitted with multiple psycho-physiological needs. This study examined the prevalence of dyspnea and the psycho-physiological factors associated with it among hospitalized Chinese patients with ILDs. We studied 165 hospitalized patients with ILDs recruited consecutively over three months in a public hospital in Guangzhou, China. Dyspnea and common psycho-physiological factors, including cough symptoms, activity capacity, lung function, physical and mental health status, and anxiety and depression symptoms, were measured. By ordered logistic regression, level of dyspnea statistically significantly affected performance in a six-minute walk test and physical functioning in work or other regular daily activities in hospitalized patients with ILDs. Respiratory rehabilitation with an appropriate intensity of exercise training or other strategies for enhancing the physical functioning of this population with moderate and severe levels of dyspnea should be prioritized.

## 1. Introduction

Interstitial lung diseases (ILDs) comprise a group of diffuse parenchymal lung diseases characterized by pulmonary interstitial inflammation and fibrosis [1]. Generally, interstitial lung diseases are chronic and progressive diseases with poor prognosis and high mortality [2]. Recently, the incidence of ILDs has been growing [3]; however, current therapies for ILDs have had limited effect on stopping the progression of the diseases [4]. Previous studies have found that an effective approach to improving psycho-physiological health and quality of life in patients with ILDs is to manage various symptoms of the diseases [5].

Dyspnea is defined as a subjective feeling of having difficulty breathing [6]. Occurring in nearly 80–90% patients with ILDs, it is the most common complaint among such patients [7,8]. Among patients with chronic respiratory diseases, including ILDs, dyspnea plays a bigger role in association with clinical outcomes than exacerbation risk or disease severity, predicting hospitalization [9], a poorer quality of life, and a shorter life expectancy [7,10,11]. Although several pharmacological and non-pharmacological therapies have been used to improve symptoms of dyspnea in patients with ILDs, researchers have very limited understanding of the influence of the psycho-physiological health of ILD patients with dyspnea [12]. Therefore, understanding the associates of dyspnea in hospitalized patients with ILDs may be of help in developing ways to effectively manage respiratory rehabilitation, and ultimately to improve quality of life and prolong the life expectancy of the patients. Earlier studies reported that both psychological factors, such as anxiety, depression, and impaired mental health status [13], and physiological abnormalities, including cough symptoms, decreased activity capacity, a decline in lung function, and damaged physical health status [7], occur with dyspnea among this group of patients. Attempts have been made to explore the association of these factors with dyspnea among sufferers with ILDs in different Western countries [7,14,15,16,17,18,19,20,21,22,23,24,25,26]. However, most of those studies have focused on the relationship between dyspnea and one or two psycho-physiological factors. Furthermore, near half of the studies were on patients with a certain subgroup of ILDs, such as idiopathic pulmonary fibrosis (IPF) [14,17,20,21,22,23], chronic hypersensitivity pneumonitis (CHP) [20], sarcoidosis [16], and connective tissue disease-related ILD [25]. Very few of the studies that have taken a more comprehensive approach have examined both the psychological and the physiological associates of dyspnea. Among the very few studies that have, the findings have been inconsistent. For example, Ryerson and colleagues [24] found that lung function, such as forced vital capacity (FVC), is not an independent determinant of dyspnea, completely contradicting the findings of another study [19]. Ryerson and colleagues found that depression was an independent predictor of dyspnea among 52 patients with ILDs. This again contradicts the findings of another study, which reported that neither anxiety nor depression was a contributor to dyspnea among patients with ILDs [8]. Therefore, a new study is needed to further clarify the relationships of both psychological and physiological factors to dyspnea in patients with various types of ILDs. Furthermore, none of the previous studies in this area were conducted among a Chinese population. Considering the effect of ethnicity on the interpretation and reporting of dyspnea [27], as well as the culturally specific descriptions of dyspnea among Chinese patients [28], it is reasonable to wonder whether the prevalence of dyspnea and associated factors in Chinese patients with ILDs might differ from those in Western patients. The purposes of this study are to: (1) describe the severity of dyspnea symptoms among hospitalized Chinese patients with ILDs; (2) investigate the association between psycho-physiological health and the level of dyspnea symptoms; and (3) explore how both the psychological and physiological factors relate to the severity of dyspnea in this population. We hypothesized that selected physiological and psychological factors are significant associates of dyspnea in a Chinese population with ILDs.

## 2. Methods

A cross-sectional study was carried out in a respiratory department of the First Affiliated Hospital of Guangzhou Medical University, Guangzhou, China. Subject recruitment was conducted consecutively from August to October 2016. All subjects gave their informed consent for inclusion before participating in the study. The study was conducted in accordance with the Declaration of Helsinki, and the protocol was approved by the Ethics Committee of the Hong Kong Polytechnic University (Ethics Approval Number: HSEARS20160615001) and the Medical Ethical Committee of the First Affiliated Hospital of Guangzhou Medical University (Ethics Approval Number: 2016-43). We included hospitalized Chinese adult patients who had been diagnosed with ILDs according to the guidelines of the American Thoracic Society (ATS) and the European Respiratory Society (ERS) [29]. We excluded patients who were unable to communicate verbally and who were not told of their illness.

Four types of data were collected on the patients during their time in the hospital: (1) their socio-demographic data and clinical characteristics; (2) their degree of dyspnea; (3) data on their physiological health; and (4) data on their psychological health.

Socio-demographic data (age, gender, marital status, educational attainment, working status prior to hospitalization, and annual family income per capita), clinical characteristics (smoking status, and duration of diagnosis with ILD), degree of dyspnea, cough symptoms, anxiety and depression symptoms, and perceived health status were assessed via a structured questionnaire completed by self-reports or interviews. Activity capacity was measured by a six-minute walk test. Other information, including on the specific types of ILDs that had been diagnosed, and lung functions, were retrieved from the patients’ medical records.

We assessed the degree of a patient’s dyspnea by using the Medical Research Council’s (MRC) dyspnea scale [30], in which the grade of dyspnea ranges from 1 to 5, with a higher score indicating more severe dyspnea. Specifically: 1, “Not troubled by breathlessness except on strenuous exertion”; 2, “Short of breath when hurrying on the level or walking up a slight hill”; 3, “walks more slowly than people of the same age on level ground because of breathlessness or having to stop for breath when walking at own pace”; 4, “has to stop for breath after walking about 100 yards or after a few minutes on level ground”; and 5, “too breathless to leave the house or breathless after undressing”. Cough symptoms were measured using a simplified cough symptom score scale containing two subscales for cough symptoms, namely, for those that occur during the daytime and those that occur during the nighttime. Possible scores for each subscale ranged from 0 (no coughing during the day/night) to 3 (frequent coughing that seriously interferes with daily activities/coughing that seriously interferes with sleep) [31]. The Hospital Anxiety and Depression Scale (HADS) was adopted for measuring anxiety and depressed symptoms [32]. The HADS consists of 14 items—seven for anxiety symptoms and seven for depression symptoms—with possible scores ranging from 0 to 21 for each of the subscales. The symptoms were divided into three categories of severity according to the scores: normal (0–7 points), borderline abnormal (8–10 points) and abnormal (11–21 points). The scale was found to have acceptable reliability and validity in hospitalized Chinese patients [33]. Physical and mental health status were examined by using the Chinese version of the 36-item short form health survey (SF-36), which comprises eight domains in two subscales that measure the health status of respondents during 4 weeks preceding to the measure [34]: the physical component summary (PCS) for physical health status, and the mental component summary (MCS) for mental health status. The eight domains included in the SF-36 are: (1) physical functioning: the extent to which an individual’s performance in physical activities such as walking, climbing, bending, etc. during a typical day are limited by health problems; (2) role-physical: the degree to which physical health problems interfere with work or other daily activities; (3) bodily pain: presence and interference of pain; (4) general health: people’s opinions about personal health; (5) vitality: energy or fatigue; (6) social functioning: problems with social activities because of physical or/and emotional problems; (7) role-emotional: the point to which emotional problems trouble work or daily activities; and (8) mental health: feelings about people’s life [35]. Each domain has a possible score range of 0 to 100, with higher scores indicating better health status. The internal consistency reliability and test-retest reliability of the SF-36 were confirmed in a previous study [36].

The six-minute walk test (6MWT) was carried out in a 24-m corridor to assess activity capacity on the basis of the guidelines of the American Thoracic Society (ATS) [37]. The results were expressed in accordance with the total distance in meters walked within six minutes, or the six-minute walk distance (6MWD).

All pulmonary function tests were performed routinely for patients with ILDs by technicians in the pulmonary function laboratory. Spirometry was used to examine the patients’ forced vital capacity (FVC) and forced expiratory volume in one second (FEV_1_), while whole-body plethysmography and the multi-breath N_2_ wash-out method was used to measure total lung capacity (TLC). The single breath and re-breathing methods were applied to assess the diffusing capacity of the lung for carbon monoxide (DLCO) (Master Screen, Hoechberg Jaeger, Germany; Quark PFT4, COSMED, Roma, Italy).

Before the present study, we carried out a pilot study to examine the reliability and validity of the measuring instruments among hospitalized Chinese patients with ILDs. Two nurses and one doctor experienced in taking care patients of with ILDs were invited to rate the content validity of the questionnaire. The same set of questionnaires was completed by 30 subjects over an interval of between 3 and 14 days to assess test-retest reliability. The test-retest reliability measured by an intra-class correlation coefficient was acceptable to excellent (>0.7) for all items in the questionnaire, with the exception of the item measuring cough symptoms during the daytime (0.63). The internal consistency reliability of the questionnaire measured by Cronbach’s alpha was >0.8, and the content validity index (CVI) measured with scale-level CVI (universal agreement) was excellent (>0.8) for all items.

## 3. Statistical Methods

A statistical analysis was performed using the Statistical Package for the Social Sciences (SPSS) software, version 23.0 (IBM Corporation, Armonk, NY, USA). We first described the overall prevalence of dyspnea and its prevalence in each of the MRC-defined dyspnea levels, in accordance with different types of interstitial lung disease. The mean and standard deviation (SD) were used to describe interval and ratio variables such as age. Frequencies and percentages were applied to describe nominal and ordinal variables such as gender. Second, Spearman’s correlation coefficient was computed for testing the associations of dyspnea with continuous variables such as the six-minute walk distance. Line-by-line association was performed to examine the association between dyspnea and ordinal variables such as cough symptoms. Last, a multivariate ordered logistic regression analysis was performed by taking the grade of dyspnea as the dependent variable, while the variables with a correlation coefficient equal or larger than 0.3 were treated as in dependent variables for dyspnea. The probability level of <0.05 was considered statistically significant. Given that the dependent variable (MRC-defined dyspnea) was a five-level categorical variable, the regression coefficients from the model have the interpretation of in odds ratios and we presented the proportional odds ratio for an easier interpretation. Specifically, for an increase in one unit in any given predictor variable, the odds of a high category (e.g., level 5) versus the combined low categories of dyspnea (e.g., levels 1–4) would be the coefficient exponentiated. Further, we adjusted the model by including other covariates, namely age, gender, smoking status and family household income.

## 4. Results

### Characteristics of the Participants

We recruited 165 patients with ILDs consecutively. They completed the questionnaires by self-reports or interviews. The characteristics of the patients are outlined in Table 1. Participants had an average age of 55.9, with 86 females and 79 males. Most were married (90.3%) at the time of data collection. Almost half of the participants had a senior high school education (49.7%) and were retired (48.5%). The annual family income per capita of more than 75% of the patients put them in the middle class or above. The patients in our study had been diagnosed with ILDs for fewer than two years (20.1 months). The most common diagnosis was diffuse parenchymal lung disease (DPLD) of known causes (34.5%), followed by idiopathic interstitial pneumonias (IIP) (21.2%), interstitial pneumonias with autoimmune features (IPAF) (9.1%), other forms of DPLD (4.2%), and granulomatous DPLD (3.6%). Apart from patients with definitive diagnoses, 45 (27.3%) patients failed to be diagnosed with a specific type of ILD. Of the 165 participants, 59 (35.8%) had once smoked but had quit by the time of the survey, with an average lifetime tobacco exposure of 30.5 pack-years.

The description of dyspnea symptoms in all patients and patients in different subgroups is shown in Table 2. Dyspnea symptoms were found in 121 (73.3%) patients, and 40% of patients assessed themselves as having grade 3, 4, or 5 dyspnea on the MRC scale, which corresponds to a moderate to severe level of dyspnea. Having compared the prevalence of dyspnea among patients in various subgroups, we found that dyspnea symptoms occurred most frequently in patients with idiopathic interstitial pneumonias (80%), followed by DPLD of known causes (71.9%), other forms of DPLD (71.4%), IPAF (66.7%), and granulomatous DPLD (33.3%). However, the difference between groups was not statistically significant, nor was the difference in the prevalence of moderate to severe dyspnea (data not shown).

Fifteen psychological (symptoms of anxiety and depression, vitality, social functioning, role-emotional, and mental health) and physiological variables (cough symptoms during the day- and nighttime, 6MWD, TLC, DLCO, physical health, role-physical, bodily pain, and general health) were found to have a statistically significant association with the severity of the dyspnea of the patients with ILDs. A descriptive statistical and bivariate analysis of the physical and psychological variables with dyspnea is summarized in Table 3. Cough symptoms in the day were more common than in the night (72.1% vs. 56.4%). More severe symptoms of coughing both during the day (line-by-line association (*df*) = 23.6 (1); *p* < 0.001) and night (line-by-line association (*df*) = 25.4 (1); *p* < 0.001) had a significant association with more severe dyspnea. The walking distance, which averaged 419 m within six minutes, had a significant correlation with the degree of dyspnea (r = −0.61; *p* < 0.001). Generally, the results of the lung function tests (percent of predicted) showed a downward trend with the increase in the degree of dyspnea. Two of the measured parameters of lung functions, including TLC (r = −0.32; *p* < 0.001) and DLCO (r = −0.52; *p* < 0.001), had a statistically significant association with dyspnea. A significant association was found between impaired physical and mental health status in all domains and the severity of the dyspnea. Of all the variables tested by Spearman’s rank correlation coefficient, the score in the physical functioning domain (r = −0.74; *p* < 0.001) had the strongest correlation with dyspnea, followed by the six-minute walk distance (r = −0.61; *p* < 0.001) and the diffusing capacity of the lung for carbon monoxide (DLCO, % predicted) (r = −0.52; *p* < 0.001). Twenty-nine (17.6%) and forty-one (24.8%) patients were at the borderline or abnormal level for anxiety or depression symptoms, respectively. Among all of the participants, twenty (12.1%) were at borderline level or met the criteria for abnormal in both anxiety and depression symptoms.

An ordered logistic regression analysis including all variables with correlation coefficients equal to and larger than 0.3 was performed to identify the factors associated with dyspnea among hospitalized Chinese patients with ILDs. Using ordered logistic regression rather than binary logistic regression allowed us to avoid dichotomizing the multilevel categorical dependent variable: degree of dyspnea. Odds ratios (95% confidence intervals) for variables predicting a higher severity of dyspnea are presented in Table 4. Two independent associates influenced by dyspnea were also identified: physical functioning (*p* = 0.004) and walk distance in six minutes (*p* = 0.046). The distance a patient walked increased by one meter, and the odds of the patient having high-level dyspnea versus having the combined low levels of dyspnea were 0.99. Similarly, for a one-point increase in the physical functioning domain, the odds of the high category versus the combined low categories of dyspnea were 0.95.

## 5. Discussion

To the best of our knowledge, this is the first study to investigate psycho-physiological association factors simultaneously (such as cough symptoms, activity capacity, lung function, anxiety, depression, and health status) with dyspnea among hospitalized Chinese patients with ILDs. Our study is consistent with previous studies that found dyspnea and impaired physiological and psychological health to be prevalent in patients with ILDs. Both physiological and psychological factors were significantly related to dyspnea in this group of patients. However, physical functioning, such as the ability to walk a given distance in six minutes, was found to be influenced more strongly than psychological functioning in terms of the degree of dyspnea. These results indicate that strategies for enhancing both physiological and psychological functioning should be adopted in hospitalized patients with ILDs. Considering the greater role of physical over psychological variables in dyspnea, interventions focusing on improving physiological functioning, such as exercise training [38] and pulmonary rehabilitation [5], could be given priority in efforts to manage dyspnea among this population.

### 5.1. Prevalence of Dyspnea

This study shows that dyspnea is a common problem (73.3%) in hospitalized Chinese patients with ILDs. Consistent with other studies, the percentage of patients with moderate to severe dyspnea (MRC grade 3 or above) in the present study was 40% compared with 35.9% among patients with an average age of 58 in France [8] and 56.4% among patients aged about 68 years in Japan [7]. In our study, no statistical difference was detected in terms of the prevalence of moderate to severe dyspnea between patients with different types of ILDs. Nevertheless, the prevalence in each subgroup was much lower than that reported in studies on patients with certain subtypes of ILDs. For example, studies in Greece have noted a higher prevalence of 60–73.1% in patients with idiopathic pulmonary fibrosis (IPF) [21,23]. However, the small sample size in the present study limited further analysis with more precise division of the ILDs patient group. Our results also indicated a lower prevalence of moderate to severe dyspnea than in Chinese patients with other respiratory diseases, such as the 50.4% reported among patients with chronic respiratory disease (COPD) [39]. Comparatively, the rate among patients with ILDs in this study was much higher than among the general population living in Guangzhou (5%) [40].

### 5.2. Physiological Factors and Dyspnea

This is the first study to examine the association between cough symptoms and dyspnea among patients with various types of ILDs. Similar to previous studies on patients with a sub-type of ILDs, such as scleroderma-ILD [41] and other chronic respiratory diseases such as asthma [42], our study found that cough symptoms were significantly correlated with dyspnea. Our study also indicated that cough symptoms at nighttime have a stronger association with dyspnea than the same symptoms during the day. This is consistent with the findings of another study on patients with gastro-esophageal reflux disease, which is a common co-morbid condition in patients with ILDs [43]. The researchers in that study found that symptoms at nighttime were likely to produce greater difficulties for the patient in dealing with his/her illness than comparable symptoms during the day. There are several findings that may explain the result. First, obstructive sleep apnea occurs frequently among individuals with ILDs, and it has been found to be significantly correlated with disease severity, including dyspnea symptoms [44]. Second, it has been reported that difficulty in falling asleep and staying asleep is accompanied by the fear of death and dying due to nighttime dyspnea in patients with COPD [45]. Cough symptoms at nighttime may compound these upsetting thoughts and, in turn, aggravate a patient’s difficulty in breathing. However, in this study, no statistically significant association was observed between dyspnea and cough symptoms either during the day or at night.

Consistent with our study, patients with ILDs were able to walk 400–500 m within six minutes in Japan [7] and Western countries [18,19]. In China, a median distance of 468 m was previously reported among patients with ILDs from a hospital outpatient department [46]. However, their performance in the 6MWT was still worse than that of healthy people. In a study of healthy Chinese participants, on average, age-matched participants were able to walk 560 m within six minutes, much further than the 419 m observed in our study [47]. In addition, they experienced inspiratory difficulties at the end of exercise more frequently than healthy people did [48]. Previous researchers have demonstrated a strong association between activity capacity (as measured by 6MWT) and dyspnea among patients with IPF (r = −0.59, *p* < 0.01; r = −0.781; *p* < 0.001; respectively) [21,26] and those with COPD (*p* = 0.03) [49]. Manali and colleagues [21] also found that 6MWD was the only variable that independently contributed to dyspnea among patients with IPF when the parameters of lung functions and the results of cardiopulmonary exercise testing were included in the regression model. The consistency of these studies strengthens the independent association between activity capacity and dyspnea. The effectiveness of exercise training for exercise performance in improving dyspnea has been proven in patients with COPD [50]. Strategies aimed at enhancing activity capacity could be effective in improving dyspnea in this patient group, and further studies should be carried out to confirm the hypothesis.

Similar to our findings, a mild decline in lung function in terms of TLC (73–76%) and a moderate decline in DLCO (44–56.1%) have been reported in other patients with ILDs [7,13,19]. Despite the weak and even non-significant correlations between dyspnea assessed by other instruments and lung functions [8,19,24], several studies have shown significant correlations between dyspnea as measured by the MRC scale and lung function. For instance, dyspnea in patients with IPF was significantly negatively related to lung function [22,23]. Another study has linked more severe dyspnea to loss of lung function, such as DLCO (r = −0.36; *p* < 0.01), in 101 patients with ILD of various categorizations [26]. Ryerson and coworkers [24] proved a weak contribution from DLCO (r = 0.05; *p* = 0.007) to dyspnea as assessed using the Baseline Dyspnea Index. In our study, TLC (percent of predicted) and DLCO (percent of predicted) were found to be significantly correlated with dyspnea, but the influence of dyspnea in the performance of lung function tests (TLC and DLCO) was not stronger than on their daily physical functioning such as walking for a short time (6 min).

Diminished physical health status has been identified in patients with ILDs in various studies. For example, in a study of Chinese patients with ILDs recruited from an out-patient department of a hospital [46], the participants’ performance in the domains of physical health status was similar to that in our study with hospitalized patients, with the lowest score found in the role-physical domain (40.66) and the highest in the bodily pain domain (75.73). Compared with patients with ILDs in the West [13,15], our patients generally obtained higher scores in physical domains such as physical functioning, which may be due to the lower age of the participants in our study [51]. In line with earlier studies [13,14,15,46], we observed a significant correlation between physical health status domains and dyspnea. Unlike previous findings that dyspnea makes a great contribution to physical health status [13,46], our study found that physical health status was of great value in predicting the degree of dyspnea. All of these results imply an interdependent relationship between dyspnea and physical health status, suggesting that strategies for improving physical functioning could be of utmost importance to patients with ILDs who have moderate and severe levels of dyspnea. This is of important and immediate significance, because physical functioning affects fundamental daily activities such as walking, bending, climbing stairs, bathing and dressing themselves on a daily basis. Previous studies have indicated that pulmonary rehabilitation, including exercise training at appropriate intensities is an effective treatment for patients with chronic diseases [52].

### 5.3. Psychological Factors and Dyspnea 

Comparing studies in Western countries with our samples of Chinese patients admitted for problems with managing ILDs [13,15], our study samples reported remarkably lower scores in several mental domains, including social functioning, role-emotional, and mental health. A number of explanations relating to cultural differences may explain the wide discrepancies in the scores. The first concerns the availability and accessibility of social and health care [53] in China, with high-quality medical resources, especially for diseases with a low incidence such as ILDs, often concentrated only in cities, giving rise to difficulties for non-urban residents in seeking medical services. Public attitudes are another possible explanation [53], with respiratory diseases often misinterpreted as communicable diseases in our society and thus hindering patients’ social activities. The last explanation concerns the anticipated stigma of chronic illness, which has been significantly associated with poor health status and physical inactivity, both prevalent in Asian countries and more common in developing countries than in developed countries [54,55]. These inconsistencies suggest that clinical workers should consider the cultural background of patients when evaluating their mental health status. Although Chang and colleagues [15] observed no statistically significant association between dyspnea and mental health status, our findings were consistent with those of other studies [13,46]. We found that all mental health-related domains (as measured by SF-36) were inversely correlated with dyspnea. The prevalence of anxiety (as measured by HADS) in our study (17.6%) was similar to the expected prevalence in Chinese patients with ILDs from an outpatient department (22.4%) [46], but below that (31%) found in another study [18]. This is probably due to the fact that the subjects in the latter study were enrolled when they were visiting a referral clinic for the first time. A lack of information and the complexity of the disease may have led to a high level of uncertainty and consequently resulted in more severe anxiety among those patients [56]. The prevalence of both clinically meaningful anxiety and anxiety at the borderline level in the current study is much higher than that in hospitalized Chinese patients with other respiratory diseases such as lung cancer (9.1% vs. 2.34%) [57] and COPD (17.6% vs. 9.6%) [58]. Similar to the high prevalence (21.9–24%) of depression reported in Beijing [46] and Australia [18], we observed a high proportion of depressed patients (as assessed by HADS) in our study. It is worth noting that 12.1% of patients with symptoms of anxiety in our study also had depression symptoms, because an increased risk of death has been identified in patients with COPD with mixed anxiety and depression symptoms [59]. Therefore, nurses should be aware of the practical importance of incorporating assessments of psychological symptoms in nursing assessment procedures, and of enhancing their sensitivity in recognizing this group of patients who are at a high risk of psychological dysfunction. The significant association between anxiety and depression symptoms (as measured by HADS) and dyspnea in our study echoed that found in an earlier study [18]. Although an independent relationship between depression and dyspnea measured by other instruments has previously been shown [24], no psychological factor was found to be able to independently predict dyspnea in our study when physical de-conditioning was considered simultaneously. This finding is confirmed by the evidence that dyspnea among individuals with ILDs [8] and people with sarcoidosis [16], which is a subtype of ILDs, is not predicted by psychological factors such as anxiety and depression. Chinese patients were more likely than Westerners to place an emphasis on somatic symptoms rather than on psychological symptoms [60], which may explain our results. In addition, instruments used both in past studies and in our study focused only on activity limitation, which may affect the presence of an association between psychological health and dyspnea. A multi-dimensional model of dyspnea, comprising an affective dimension and a sensory dimension, has recently been proposed [61]. The ATS has also suggested that dyspnea can be evaluated in three different domains [62]. Given the limited number of related studies and the contradictory results, the value of psychological health in prognosticating the severity of dyspnea remains open to discussion, with a disease-specific and more comprehensive assessment of dyspnea awaiting future study.

Our study has several limitations. First, the cross-sectional nature of the study precluded us from inferring a causal relationship between the selected variables and dyspnea. Second, the uni-dimensional tool for measuring dyspnea cannot reflect different aspects of dyspnea symptoms. Third, the small sample size and the high percentage of patients with no specific diagnosis limited the analysis of subtypes; it is possible that the related factors of dyspnea and the associations differed among patients with different types of ILDs. Fourth, the results of the study may have limited its generalizability because the subjects were only recruited from one city. However, the hospital that hosted our study is the largest institution for respiratory diseases in south China, so the participants in our study, coming as they did from various parts of the country, have a certain representativeness. Fifth, some data were missed because some of the patients did not participate in the six-minute walk test and/or did not complete the lung function tests due to their poor physical condition. Finally, not all potential contributors, such as other co-morbid conditions, arterial oxygen saturations (SaO_2_)—basal and during the 6MWT—psychological factors such as dyspnea-related fear, and treatment for dyspnea symptoms, were collected in our study.

## 6. Conclusions

In conclusion, the present study suggests that, in hospitalized Chinese patients with ILDs, dyspnea is a very common problem and has a considerable impact on their lives. Clinical workers should take dyspnea into account when evaluating the outcomes of care for individuals with ILDs, because of the wide range of aspects it involves. In clinical practice, treatment strategies for ILDs are commonly based on disease severity, which is detected by objective measurements. However, our findings suggest that factors related to personal perceptions, such as physical functioning and actual performance in terms of distance walked in six minutes, were significantly influenced by the degree of dyspnea in this group of patients. Patients with impaired physical functioning find it difficult to perform many activities of daily living. Therefore, interventions aimed at improving their exercise capacity should be promoted. A longitudinal assessment of the association between physiological and psychological factors and dyspnea is required to substantiate the current findings.

## Figures and Tables

**Table 1:** Characteristics of the participants (*n* = 165).

Characteristics	Mean ± SD or *n* (%)
Age, years	55.9 ± 13.0
Male gender	79 (47.9)
Marital status	
Married	149 (90.3)
Never married	5 (3.0)
Divorced	1 (0.6)
Widowed	10 (6.1)
Education	
Primary school and below	40 (24.2)
Junior high school	43 (26.1)
Senior high school	42 (25.5)
College and beyond	40 (24.2)
Working status prior to hospitalization	
Working full-time	42 (25.5)
Working part-time	15 (9.1)
Not working	28 (17.0)
Retired	80 (48.5)
Family income per capita *	
<US$2219	40 (24.2)
US$2219~$5547	58 (35.2)
>US$5547	67 (40.6)
Duration since ILD was diagnosed ** (month)	20.1 ± 24.1
Types of ILDs	
Diffuse parenchymal lung disease of known cause	57 (34.5)
Connective tissue disease-related ILD	55 (33.3)
Drug-induced ILD	1 (0.6)
Environmental exposure-related ILD	1 (0.6)
Idiopathic interstitial pneumonias	35 (21.2)
Idiopathic pulmonary fibrosis	16 (9.7)
Unclassifiable idiopathic interstitial pneumonias	8 (4.8)
Idiopathic nonspecific interstitial pneumonia	7 (4.2)
Idiopathic lymphoid interstitial pneumonia	3 (1.8)
Cryptogenic organizing pneumonia	1 (0.6)
Interstitial pneumonias with autoimmune features	15 (9.1)
Other forms of diffuse parenchymal lung disease	7 (4.2)
Pulmonary Alveolar Proteinosis	5(3)
Idiopathic pulmonary hemosiderosis	1 (0.6)
Giant cell interstitial pneumonia	1 (0.6)
Granulomatous diffuse parenchymal lung disease	6 (3.6)
Hypersensitivity pneumonitis	4 (2.4)
Sarcoidosis	2 (1.2)
Unclassified	45 (27.3)
Smoking Status	
Never Smoked	106 (64.2)
Formerly Smoked ***	59 (35.8)
Currently Smoking	0 (0)
Pack-years **** Smoked of Former Smoker	30.5 ± 24.7

ILDs: interstitial lung diseases; * converted at the following exchange rate: US $1 = ¥6.76; ** rounded down in a complete month; *** person who smoked ≥100 cigarettes over their lifetime; **** pack-years = the number of cigarettes smoked per day/20 × the number of years smoked.

**Table 2:** Description * of dyspnea in patients with ILDs.

Diagnoses (*n*)	Dyspnea ^a^					Prevalence of Grade 3–5 Dyspnea ^b^	Overall Prevalence by All Sub-Types of ILDs
	Grade 1	Grade 2	Grade 3	Grade 4	Grade 5		
Mix of ILDs (*n* = 165)	44 (26.7)	55 (33.3)	40 (24.2)	16 (9.7)	10 (6.1)	66 (40.0)	121 (73.3)
DPLD of known Cause (*n* = 57)	16 (28.1)	19 (33.3)	12 (21.1)	6 (10.5)	4 (7.0)	22 (38.6)	41 (71.9)
IIP (*n* = 35)	7 (20.0)	16 (45.7)	8 (22.9)	2 (5.7)	2 (5.7)	12 (34.3)	28 (80.0)
IPAF (*n* = 15)	5 (33.3)	2 (13.3)	5 (33.3)	2 (13.3)	1 (6.7)	8 (53.3)	10 (66.7)
Other forms of DPLD (*n* = 7)	2 (28.6)	2 (28.6)	3 (42.9)	0 (0)	0 (0)	3 (42.9)	5 (71.4)
Granulomatous DPLD (*n* = 6)	4 (66.7)	1(16.7)	1 (16.7)	0 (0)	0 (0)	1 (16.7)	2 (33.3)

* Data were presented as frequency (%); ^a^ 1 = “Not troubled by breathlessness except on strenuous exertion”; 2 = “Short of breath when hurrying on the level or walking up a slight hill”; 3 = “walks slower than people of the same age on level ground because of breathlessness or having to stop for breath when walking at own pace”; 4 = “has to stop for breath after walking about 100 yards or after a few minutes on level ground”; and 5 = “too breathless to leave the house or breathless after undressing”. ^b^ moderate to severe dyspnea; ILDs, interstitial lung diseases; DPLD, diffuse parenchymal lung disease; IIP, Idiopathic interstitial pneumonias; IPAF, interstitial pneumonias with autoimmune features.

**Table 3:** Association of physiological and psychological factors with dyspnea.

Variables *	Level of Dyspnea ^a^						Rho ^#^/r(*df*) ^##^
	Grade 1	Grade 2	Grade 3	Grade 4	Grade 5	All Patients	
Cough symptoms							
Daytime							3.6 (1) ^†††^
No cough during the day	22 (47.8%)	13 (28.3%)	9 (19.6%)	1 (2.2%)	1 (2.2%)	46 (27.9%)	
Occasional cough for short periods	20 (20.8%)	35 (35.5%)	26 (27.1%)	10 (10.4%)	5 (5.2%)	96 (58.2%)	
Frequent cough that mildly interferes with daily activities	2 (10.5%)	7 (36.8%)	5 (26.3%)	3 (15.8%)	2 (10.5%)	19 (11.5%)	
Frequent cough that seriously interferes with daily activities	0 (0.0%)	0 (0.0%)	0 (0.0%)	2 (50.0%)	2 (50.0%)	4 (24.2%)	
Nighttime							25.4 (1) ^†††^
No cough during the night	29 (40.3%)	26 (36.1%)	14 (19.4%)	2 (2.8%)	1 (1.4%)	72 (43.6%)	
Cough for short periods when falling asleep, or occasional cough at night	14 (18.9%)	23 (31.1%)	22 (29.7%)	9 (12.2%)	6 (8.1%)	74 (44.8%)	
Cough that mildly interferes with sleep	0 (0.0%)	5 (45.5%)	3 (27.3%)	3 (27.3%)	0 (0.0%)	11 (6.7%)	
Cough that seriously interferes with sleep	1 (12.5%)	1 (12.5%)	1 (12.5%)	2 (25.0%)	3 (37.5%)	8 (4.8%)	
Activity capacity (*n* = 147)							
6MWD(m)	491 (75)	432 (70)	380 (91)	307 (67)	150 (39)	419 (101)	−0.61 ^†††^
Lung function, percent pred							
TLC ^b^ (*n* = 123)	77.6 (15.4)	67.3 (14.6)	69.7 (15.4)	47.5 (22.3)	45.3 (na)	69.8 (17.0)	−0.32 ^†††^
FEV1/FVC ^c^ (*n* = 143)	99.4 (10.3)	104.9 (8.7)	100.4 (15.2)	106.4 (11.6)	105.9 (4.6)	102.2 (11.5)	0.16
DLCO ^b^ (*n* = 133)	64.5 (15.7)	52.1 (15.3)	43.8 (13.4)	41.4 (11.5)	24.0 (na)	52.9 (17.0)	−0.52 ^†††^
SF-36, component scores and domain scores ^d^							
Physical Functioning	89.4 (10.1)	75.5 (15.6)	64.5 (18.9)	43.8 (18.6)	8.5 (8.5)	69.4 (25.4)	−0.74 ^†††^
Role-Physical	61.4 (41.2)	44.5 (38.4)	22.5 (32.4)	10.9 (27.3)	0.0 (0.0)	37.7 (40.4)	−0.50 ^†††^
Bodily Pain	80.1 (24.9)	77.1 (23.3)	68.6 (28.3)	73.8 (29.4)	50.3 (39.3)	73.9 (27.4)	−0.19 ^†^
General Health	51.9 (18.5)	46.1 (19.7)	34.9 (18.0)	28.1 (16.7)	22.9 (16.1)	41.8 (20.5)	−0.45 ^†††^
Vitality	72.6 (14.2)	66.7 (17.9)	49.8 (23.3)	56.9 (15.5)	34.0 (24.7)	61.2 (21.6)	−0.44 ^†††^
Social Functioning	76.7 (23.0)	70.0 (21.1)	60.3 (21.5)	56.3 (26.2)	36.3 (30.9)	66.1 (24.8)	−0.37 ^†††^
Role-Emotional	72.7 (36.1)	54.5 (40.2)	38.3 (37.4)	43.8 (33.8)	33.3 (35.1)	53.1 (39.6)	−0.36 ^†††^
Mental Health	77.2 (13.7)	75.0 (16.9)	65.8 (19.9)	63.0 (20.0)	49.2 (28.0)	70.6 (19.3)	−0.31 ^†††^
HADS, subscale scores							
Anxiety ^e^	3.6 (2.8)	3.5 (2.7)	6.0 (4.2)	5.1 (4.1)	8.9 (5.7)	4.6 (3.8)	0.24 ^††^
Depression ^e^	2.9 (2.4)	4.4 (3.5)	6.5 (4.7)	6.0 (4.9)	8.7 (4.5)	4.9 (4.1)	0.36 ^†††^

* Descriptive data were expressed in terms of frequency (*n*) and percentage (%) or mean and standard deviation ^#^ Spearman’s correlation coefficient; ^##^ line-by-line association; ^a^ 1 = “Not troubled by breathlessness except on strenuous exertion”; 2 = “Short of breath when hurrying on the level or walking up a slight hill”; 3 = “Walks slower than people of the same age on level ground because of breathlessness or having to stop for breath when walking at own pace”; 4 = “Has to stop for breath after walking about 100 yards or after a few minutes on level ground”; and 5 = “too breathless to leave the house or breathless after undressing”, ^b^ mild decrease (60–79%); moderate decrease (40–59%); severe decrease (<40%), ^c^ >80%:normal; <70%: obstructive ventilation disorder; ^d^ possible score ranges from 0 to 100, the higher score meaning the better the condition; ^e^ patients scored 8 or above; 6MWD, six-minute walk distance; TLC, total lung capacity; FVC, forced vital capacity; FEV_1_, forced expiratory volume in one second; DLCO, diffusing capacity of the lung for carbon monoxide; SF-36, the 36-item short form health survey; and HADS, the hospital anxiety and depression scale; *n* = number of subjects; na = not available as only one subject was involved in the cell; ^†^ *p* < 0.05, ^††^ *p* < 0.01, ^†††^ *p* < 0.001.

**Table 4:** Results of Ordered Logistic Regression Analysis (MRC grade as the dependent variable) (*n* = 116).

Associates	Unadjusted Model			Adjusted Model *		
	Odds Ratio (Exp Coefficient)	95% CI	*p*-Value	Odds Ratio (Exp Coefficient)	95% CI	*p*-Value
Cough symptoms at daytime						
Frequent cough that mildly interferes with daily activities	2.86	0.55–14.76	0.209	1.30		0.141
Occasional cough for short periods	1.93	0.63–5.90	0.247	0.54		0.354
No cough during the day	1.00	--	--	1.00	--	--
Cough symptoms at nighttime						
Cough that seriously interferes with sleep	1.92	0.16–23.72	0.610	0.44		0.745
Cough that somewhat interferes with sleep	2.19	0.41–11.76	0.362	1.46		0.134
Cough for short periods when falling asleep, or occasional cough at night	1.93	0.71–5.24	0.199	0.64		0.224
No cough during the night	1.00	--	--	1.00	--	--
6-min walk distance	0.99	0.99–1.00	0.036	0.99	0.99–1.00	0.046
TLC	0.98	0.95–1.00	0.201	0.98	0.95–1.01	0.299
DLCO	0.97	0.94–1.00	0.097	0.97	0.94–1.01	0.098
SF-36 domains						
Physical Functioning	0.95	0.92–0.99	0.005	0.95	0.92–0.98	0.004
Role-Physical	0.99	0.98–1.01	0.544	0.99	0.98–1.01	0.549
General Health	1.00	0.98–1.02	0.861	0.99	0.98–1.01	0.556
Vitality	0.99	0.96–1.02	0.391	0.99	0.96–1.02	0.478
Social Functioning	0.99	0.97–1.01	0.503	0.99	0.97–1.01	0.432
Role-Emotional	1.01	1.00–1.03	0.169	1.01	1.00–1.03	0.090
Mental Health	1.00	0.96–1.03	0.831	1.00	0.96–1.03	0.867
HADS Depression	1.04	0.91–1.21	0.512	1.06	0.91–1.24	0.420
Model summary	−2 Log Likelihood = 203.89, chi-square = 91.46, *p* < 0.001			−2 Log Likelihood = 197.07, chi-square = 98.29, *p* < 0.001		
Test of Parallel Lines	−2 Log Likelihood = 179.66, chi-square = 24.23, *p* = 0.998			−2 Log Likelihood = 165.64, chi-square = 31.43, *p* = 1.00		

* Model adjusted for age, gender, marital status, smoking status and family income; TLC, total lung capacity; DLCO, diffusing capacity of the lung for carbon monoxide; SF-36, the 36-item short form health survey; HADS, hospital anxiety and depression scale; CI, confidence interval; exp, exponential.
